# The first structure of HIV-1 gp120 with CD4 and CCR5 receptors

**DOI:** 10.1186/s13578-018-0267-6

**Published:** 2019-01-03

**Authors:** Yongjun Guan

**Affiliations:** Antibody BioPharm, Inc, 401 Professional Dr. Ste241, Gaithersburg, MD 20879 USA

**Keywords:** gp120, CCR5, Cryo-EM, HIV-1, ADCC, Trogocytosis

## Abstract

Shaik et al. recently published online the cryo-electron microscopy structure of HIV-1 gp120 in complex with CD4 and CCR5 receptors. This is the first structure of the ternary HIV-1 gp120/CD4/CCR5 complex. This breakthrough of Env structure provides insights into HIV-1 fusion mechanism, CCR5 function, co-receptor switch, and, most importantly, the development of co-receptor-targeted therapeutic inhibitor and HIV-1 vaccine. It also shed lights on the immunogenicity of gp120 by revealing the stably exposed conserved gp41-interactive region of gp120 in the complex.

## Main text

HIV-1 Env, a trimer of gp120–gp41 heterodimers, is the sole target of broadly neutralizing antibodies of which is believed to be a primary goal of HIV-1 vaccine development [1]. Env mediates membrane fusion after binding CD4 receptor and a co-receptor CCR5/CXCR4. Based on rich structural information of Env with CD4 and with antibodies, it is well recognized that Env likes a conformational machine by transitioning between conformations of prefusion-closed, CD4-bound, and co-receptor-bound into a postfusion state to facilitate virus/target cell membrane fusion. The initial CD4 receptor binding displaces V1V2 loops from the Env’s apex, allows co-receptor binding and opens up Env to enable gp41-mediated membrane fusion [2]. However, a structure of Env with co-receptor had been missing and leaved a gap in our knowledge of the interaction between Env and co-receptor..

In a recent online article of Nature, Shaik et al. solved the cryo-electron microscopy (cryo-EM) structure of a full-length monomeric gp120 in complex with a soluble CD4 ectodomain and an unmodified human CCR5 reconstituted into a lipid micelle [3]. This is the first structure of the ternary HIV-1 gp120/CD4/CCR5 complex and a breakthrough in the field. It adds a wealth of structural information on previous complexes of gp120 with CD4 and antibodies regarding the conformation of gp120, CCR5 and the interactions between gp120 and CCR5. The interpretation of the structure, as the authors addressed, provides insights into HIV fusion mechanism, CCR5 function, co-receptor switch, and, most importantly, the development of co-receptor-targeted therapeutic inhibitor and HIV-1 vaccine.

The structure revealed details of the interactions between CCR5 and gp120. It showed that the V3 loop with its conserved tip inserts into the CCR5 binding pocket, similar to that observed with the CCL5, and an extended CCR5N terminus interacts with the gp120 bridging sheet. Most importantly, the structure of the interactions between CCR5 and gp120 indicates that maraviroc works by directly competing with the binding of gp120 to CCR5, which contrasts with the view in the field that maraviroc is an allosteric inhibitor. Based on the observation that the gp120 binding site on CCR5 mainly overlaps with maraviroc in the minor subpocket, the authors suggested a new design of more powerful therapeutic drugs by “adding additional groups to the triazole ring of maraviroc to enhance its competing potency with the V3 loop”.

The overall structural of gp120 in the complex with CD4 and CCR5 is very similar to the CD4-bound structural of Env [2]. This surprising observation led the authors to imply that the co-receptor binding does not induce major conformational change of gp120 and the role of CCR5 binding is to stabilize the CD4-bound conformation of Env and to hold it closer to the cell membrane. However, the structure might represent a postfusion state of monomeric gp120 bound to CD4 and CCR5, therefore it may not reveal the changes induced by CCR5 binding in the complex of membrane anchored CD4, CCR5 and Env trimer structures. The observed difference that the N-terminus of the CCR5-bound gp120 flips back onto the 7-stranded sheet of gp120 inner domain is mostly a late-entry structure as previously revealed for the epitope structure of the cluster A C11-like antibody N12-i3 [4].

One remarkable highlight of the article is that the authors were able to obtain a high-resolution cryo-EM structure of the gp120-CD4/CCR5 complex. This technical success reflects an important feature of the gp120-CD4/CCR5 complex that the complex can be stably extracted from target cell membrane. This feature shows that the gp41-interactive region of gp120 in the late viral entry Env structure is well exposed on target cell membrane and, therefore, is highly vulnerable to antibody immune response. The gp41-interactive region of gp120 is mainly comprised conserved epitopes for the cluster A antibodies including A32-like, A32/C11 and C11-like antibodies [4]. Therefore, the article also shed an important light in immune response against Env in HIV infection and Env vaccine.

Antibodies specific for the cluster A epitopes has been shown to be common and induced early in infection, and may mediate the majority of ADCC activities detected in chronically infected individuals [5]. They were also implicated in protection for the clinical RV144 vaccine trial [6]. However, the role of cluster A antibodies is still not defined. The epitopes of cluster A antibodies are strictly receptor-binding dependent. They are not exposed on native trimer of HIV-1 viruses or productively infected cells [7]. Therefore, the antibodies may not have a direct impact on virus and productively infected cells. Further, a high throughput “rapid and fluorometric” antibody-dependent cellular cytotoxicity (RFADCC) assay was commonly used to measure ADCC activity of cluster A antibodies [8]. The RFADCC assay has been recently revealed to be actually measuring majority an antibody-dependent trogocytosis phenomenon instead of the assumed cell killing function of ADCC [9, 10], which has imposed confusing results for the role of cluster A antibodies. Cluster A antibody mediated Trogocytosis will probably remove the well exposed antibody-gp120 complexes from infected target cell surface by monocytes and may result in escape of infected cells from the immune surveillance. Therefore, it should be cautious for Env-based vaccine development targeting cluster A antibody responses before a conclusive protective role of cluster A antibody is evidenced.

In summary, the first structure of HIV-1gp120 with CD4 and CCR5 revealed by Shaik et al. is a remarkable progress in the field. The author made import insights into HIV-1 fusion mechanism, function of CCR5, co-receptor switch, as well as development of of novel inhibitor and vaccine design. It also shed light on immune response against the conserved immunodominant cluster A region of gp120.

## Abbreviations

cryo-EM: cryo-electron microscopy
RFADCC: “rapid and fluorometric” antibody-dependent cellular cytotoxicity
